# Genomic Evaluation in Nellore Cattle for Reproductive Traits: Multiple Ways to Account for Missing Pedigrees

**DOI:** 10.1111/jbg.12947

**Published:** 2025-06-06

**Authors:** Larissa Temp, Gabriel Gubiani, Ludmilla Brunes, Claudio Magnabosco, Fernando Bussiman, Jorge Hidalgo, Daniela Lourenco, Fernando Baldi

**Affiliations:** ^1^ Departamento de Zootecnia Universidade Estadual Paulista (UNESP) Jaboticabal São Paulo Brazil; ^2^ Centro de Desempenho Animal Embrapa Cerrados Planaltina Federal District Brazil; ^3^ Department of Animal and Dairy Science University of Georgia Athens Georgia USA

**Keywords:** accuracy, *Bos indicus*, metafounders, ssGBLUP, unknow parent groups

## Abstract

Missing pedigrees are a common problem in most populations. Animals with unknown ancestors are usually treated as founders; however, this can underestimate inbreeding, not properly account for different base populations, and bias breeding values. We aimed to assess the use of unknown parent groups (UPG) or metafounders (MF) to model missing pedigrees in a beef cattle population. Phenotypic and genotypic data from the Nellore improvement programme of the Brazilian Breeders and Researchers Association were used. The pedigree contained 3.8 M animals born between 1970 and 2022, of which 51,752 were genotyped. Records for scrotal circumference at 365 days old (SC365, *N* = 239,806), age at first calving (AFC, *N* = 560,785) and accumulated cow productivity (ACP, *N* = 269,330) were used. Four models were implemented: single‐step GBLUP without explicitly dealing with missing pedigree (G0), with UPG (G1), with MF (G2) and with G accounting for group‐specific allele frequencies (G3). UPG and MF were assigned based on commercial and registered herds (S1), uncertain paternity (S2) and patriarchs (S3). The accuracy and bias of predictions were assessed using the linear regression (LR) method. Linear, single‐trait animal models were used for SC365 and AFC, and multi‐trait for ACP. Heritability estimates ranged from 0.07 to 0.40. Compared to G0, accuracy was slightly higher in G2_S2_ and G2_S3_ (0.70 vs. 0.71) for SC365, G2_S3_ (0.49 vs. 0.51) for AFC, G1_S2_ for ACP (0.67 vs. 0.71). Bias was small in all the scenarios (≤ 0.06 SD), except of ACP that presented a great bias, including MF. Overall, G1 and G2 had similar accuracy, possibly because of the limited number of genotyped animals linked to MF. Centring the genomic relationship matrix by patriarchs' allelic frequencies resulted in similar accuracy and bias to the MF models. Replicating the study with a larger database containing more genotyped animals connected to MF could help improve the MF estimates, and thus, prediction accuracy and bias.

## 1 Introduction

Missing pedigrees are common in livestock populations. Animals whose parents are missing are treated as unrelated, non‐inbred founders of the population. These animals have an expected breeding value of zero, regardless of their generation in the pedigree. However, animals with unknown parents come from different generations and refer to different base populations (Tsuruta et al. 2011; Kluska et al. 2018). Assuming animals with missing pedigrees are founders is a wrong assumption that leads to underestimation of inbreeding and additive relationships. This results in incompatibility between pedigree and genomic relationships in single‐step GBLUP models and, ultimately, biased predictions (Legarra et al. 2015).

For pedigree‐based models, Quaas (1988) proposed to replace unknown parents for group effects (also known as unknown parent groups; UPG), able to account for the change in the genetic trend that is not accounted for by incomplete pedigree relationships. In practice, UPG can be defined based on birth year, sex and breed composition and proven to alleviate the bias of pedigree‐based breeding values (Legarra et al. 2007). Nowadays, single‐step GBLUP (Legarra et al. 2009; Aguilar et al. 2010) is the most used method for breeding values prediction because of its flexibility to incorporate genotyped and non‐genotyped animals in a combined relationship matrix (H). The UPG methodology may not eliminate the bias in breeding values derived from single‐step GBLUP models due to differences between the pedigree and genomic relationships (Masuda et al. 2022). Furthermore, the UPG are assumed to be unrelated as this theory was derived under pedigree‐based relationships (Legarra et al. 2015).

The UPG have been incorporated into single‐step GBLUP through a modification of $H^{-1}$ and in some cases have decreased the bias and inflation of GEBV (Tsuruta et al. 2011; Masuda et al. 2021). However, in some situations, incompatibility between the genomic (G) and pedigree (A) relationship matrices can result in biased predictions (Misztal and Legarra 2017). Because not all animals in the pedigree are genotyped, and to solve the incompatibility, the matrices need to refer to the same base population. Legarra et al. (2015) proposed the metafounders (MF) theory that generalises and extends the concept of UPG. The main idea is to modify A to ensure compatibility with G. The MF are fictitious individuals treated as both the father and mother of the base population, having within and across relationships derived from genomic information, facilitating the handling of common ancestry among individuals who would otherwise appear unrelated based on pedigree information.

A fundamental assumption of the MF approach is the construction of G with allele frequencies equal to 0.5, as stipulated by Christensen (2012), and their utility has been demonstrated across various datasets, including those in livestock (Koivula et al. 2021), sheep (Granado‐Tajada et al. 2020), pig breeding (Xiang et al. 2017) and simulated data in dairy cattle (Garcia‐Baccino et al. 2017).

Another strategy to enhance the compatibility between matrices G and A and alleviate bias and dispersion was devised by Lourenco et al. (2016); in this approach, breed‐specific (or group‐specific) allele frequencies are used to construct G. This matrix is centred to fix the mean values of the allele effects to zero, and it is adjusted to the specific allele frequencies of each founder breed, providing greater consistency between the coefficients of relationship of genotyped and non‐genotyped individuals (Makgahlela et al. 2013).

Using tools capable of increasing accuracy and reducing the bias of genomic evaluations in young animals is of utmost importance to achieve a more significant response to selection, as well as being effective strategies for reducing genotyping and phenotyping costs. Studies with MF, UPG and group‐specific **G** in Nellore beef cattle for traits related to reproduction, longevity and production are still scarce in the literature. Our aim in this study was to evaluate the effect of using one or multiple MF and UPG in predicting the estimated genomic breeding values using single‐step GBLUP and verify the impact of building **G** with patriarch‐specific allele frequencies on genomic predictions for SC365, AFC and ACP in the Nellore breed.

## 2 Material and Methods

### 2.1 Data

Phenotypic and genotypic data from the Nellore improvement program of the Brazilian Breeders and Researchers Association (ANCP) were used. ANCP provided us with records for scrotal circumference (SC365), age at first calving (AFC) and accumulated cow productivity (ACP). Because data were obtained from existing databases, the approval of the Animal Care and Use Committee was not needed for this study. SC365 was measured in centimetres with a measuring tape, from 9 to 18 months of age in intervals of 3 months, followed by linear adjustment for 365 days. AFC was simply the age in months at the cows' first calving. ACP represents the average calf weaning weight per year per cow, calculated as in Lôbo et al. (2000):

(1) $$\mathrm{ACP}=\frac{\mathrm{WW}\times n_{c}\times \mathrm{Ca}}{\mathrm{ALC}-\mathrm{Ci}}$$

where WW is the mean calf weaning weight (kg), $n_{c}$ the total number of calves produced, Ca is the constant equal to 365 days to express fertility on an annual basis, ALC is the age of the cow at last calving (days) and Ci is the constant equal to 450 days, reflecting the expectation that first calving will occur at 30 months of age.

The pedigree contained information on 3,835,379 animals born between 1970 and 2022. For all traits, contemporary groups (CG) were created by concatenating farm, birth year, birth season and weaning management group classes. The CG with less than five records was removed from the dataset. Additionally, any phenotypic records (within CG) deviating from CG mean ± 3 standard deviations were also removed. A total of 51,752 animals were genotyped with a panel containing 65,436 single‐nucleotide polymorphisms (SNP) (CLARIFIDE Nelore 4.0). Genotype quality control excluded SNPs monomorphic, minor allele frequency (MAF) less than 5%, call rate less than 90% (SNPs & animals) and parent‐progeny conflicts (SNPs & animals) performed using preGSF90 software (REF), leaving 35,871 markers for subsequent analyses. These evaluations were standardised by subtracting the mean EBV of animals born in 2015 (highest number of animals with phenotypes and genotypes available) from all breeding values. A summary of the data descriptive statistics is presented in Table 1.

**TABLE 1:** Number of phenotypic records (*N*), mean, standard deviation (SD), minimum and maximum for scrotal circumference at 365 days (SC365), age at first calving (AFC) and accumulated cow productivity (ACP) traits in Nellore breed.

Traits	*N*	Mean (SD)	Minimum	Maximum
SC365 (cm)	239,806	21.16 (2.69)	9.5	34.6
AFC (months)	560,785	35.86 (6.09)	21	49
ACP (kg/year)	269,330	148.20 (33.95)	43	466

### 2.2 Statistical Model for Variance Components Estimation and Breeding Values Prediction

A linear model was used to analyse SC365, AFC and ACP, considering fixed effects of CG and random additive animal effects. Under matrix notation, this model can be written as:

(2) y=Xβ+Zu+e

where: y is the vector of phenotypic observations (SC365, AFC or ACP); β is the vector of fixed effects (CG); u is the vector of random additive animal effects; e is the vector of random residuals; X is the incidence matrix relating the effects in β to observations in y; and Z is the incidence matrix relating the levels in u to observations in y.

Under the multivariate normality assumption, the (co)variance matrix for the random effects is given by:

(3) $$\mathrm{Var}\left[\begin{array}{c} u\\ e \end{array}\right]=\left[\begin{array}{cc} A\sigma_{u}^{2} & 0\\ 0 & I\sigma_{e}^{2} \end{array}\right]$$

where: A is the pedigree‐based relationship matrix; I is an identity matrix of proper order; $\sigma_{u}^{2}$ is the additive genetic variance for the trait being analysed and $\sigma_{e}^{2}$ is the residual variance. $\sigma_{u}^{2}$ and $\sigma_{e}^{2}$ for SC365, AFC and ACP were obtained through average information restricted maximum likelihood using the BLUPF90+ software (Misztal et al. 2014; Lourenco et al. 2022) without genomic information. Single‐trait analyses were conducted for SC365 and AFC, while a multi‐trait analysis was performed for ACP using SC365 and AFC as a base. The objective was to enhance the prediction of ACP by leveraging the larger amount of information available for the other two traits. The multi‐trait analysis aimed to benefit ACP, a trait selectively measured in females and correlated with SC365 and AFC, which are measured earlier in the animals and have more data.

### 2.3 Genomic Predictions

For all traits, four predictions were obtained: 1) regular ssGBLUP (G0); 2) ssGBLUP with MF (G1); 3) ssGBLUP with UPG as fixed effects (G2) and 4) regular ssGBLUP but G was constructed using patriarch‐specific allele frequencies (G3).

Without UPG or MF (G0), the covariance structure among animals (**H**) in ssGBLUP was as in Aguilar et al. (2010) and the **G** matrix was constructed according to Vanraden (2008).

With MF (G1), allele frequencies were assumed to be 0.5 to centre **G** (Christensen 2012), and **A** was modified with a matrix Γ whose entries are relationship coefficients among MF. Legarra et al. (2015) when coining the term MF as a pseudo‐individual that allows for different averages in the base population, it showed that 0.5 allele frequencies are a natural choice, because allelic frequencies can be seen as nuisance parameters and therefore can be marginalised (Garcia‐Baccino et al. 2017). Going back to Christensen (2012) results, the **A**, considering a ‘related base’ is $A^{\gamma }=I\left(1-\frac{\gamma }{2}\right)+J\gamma$ and **G** should be constructed as $G=\frac{ZZ^{\prime }}{s}$ where **Z** contains values $\left\{-1,0,1\right\}$ (i.e., p=0.5). What Legarra et al. (2015) proposed was an extension of Christensen's results allowing for multiple base populations, and considering the $H^{-1}$ matrix we would have:

(4) $${\left(H^{\Gamma }\right)}^{-1}={\left(A^{\Gamma }\right)}^{-1}+\left[\begin{array}{cc} 0 & 0\\ 0 & G_{05}^{-1}-{\left(A_{22}^{\Gamma }\right)}^{-1} \end{array}\right]$$

where $A^{\Gamma }$ and $A_{22}^{\Gamma }$ are equivalent to $A^{-1}$ and $A_{22}^{-1}$ modified by Γ, a positive‐definite matrix given by:

(5) $$\Gamma =\left[\begin{array}{cccc} \gamma_{1} & \gamma_{1,2} & \cdots & \gamma_{1,n}\\ \gamma_{2,1} & \gamma_{2} & \cdots & \gamma_{2,n}\\ \vdots & \vdots & \ddots & \vdots \\ \gamma_{n,1} & \gamma_{n,2} & \cdots & \gamma_{n} \end{array}\right]$$

where $\gamma_{i}$ is the relationship within the *i*th MF and $\gamma_{i,j}$ is the relationship between the *i*th and *j*th MF. Γ was estimated using pedigree and genomic information by generalised least‐squares according to Garcia‐Baccino et al. (2017).

When UPG were considered (G2), the altered Quaas‐Pollak (QP) transformation (Tsuruta et al. 2019; Masuda et al. 2022) was used to consider groups in **A** and in **G**. In this case, $H^{-1}$ is given by:

(6) $$H^{*}=A^{*}+\left[\begin{array}{ccc} 0 & 0 & 0\\ 0 & G^{-1}-A_{22}^{-1} & -\left(-A_{22}^{-1}\right)Q_{2}\\ 0 & -Q\prime_{2}\left(-A_{22}^{-1}\right) & Q^{\prime }\left(-A_{22}^{-1}\right)Q_{2} \end{array}\right]$$

where $A^{*}$ is the inverse of the additive relationship matrix based on QP transformation; $G^{-1}$ is the inverse of the genomic relationship matrix, constructed based on method I of Vanraden (2008); $A_{22}^{-1}$ is the inverse of the pedigree relationship matrix among genotyped animals; and Q is a matrix relating genotyped animals with UPG. UPG and MF were assigned based on missing pedigrees from commercial and registered herds (S1), if the animals had one or both parents' unknown (S2), and patriarchs (animals born in Brazil, sons of imported animals) (S3). In Brazil, registered animals are evaluated together with commercial (non‐registered) animals, as commercial herds often utilise genetics from registered herds. This practice promotes genetic connectivity between the two populations. Ten patriarchs were chosen based on ANCP producers' demand, who are more likely to use bulls descended from the first animals brought from India to Brazil, and who have a greater influence on genetic evaluations. These patriarchs are descendants of Nellore breed lineages imported from India to Brazil, primarily originating from the Ongole region in Andhra Pradesh. The importation, which began in 1870 during the colonial period, included highly influential bulls such as Kavardi, Taj Mahal, Kurupathy, Golias, Godhavari and Rastã (Magnabosco et al. 1997). A summary of the evaluation scenarios is presented in Table 2.

**TABLE 2:** Scenarios tested for longevity and reproductive traits in the evaluation of Nellore breed using UPG and MF.

	Definition
G0	G default with the current allele frequencies, constructed as Vanraden (2008)
G3	G matrix was centred and scaled by MF patriarchs specific allele frequencies as in Lourenco et al. (2016)
S1	Commercial and registered herd
S2	Paternity: both parents unknown and just one of the parents is unknown
S3	Patriarchs: Ludy de Garça, 1646 da M.N., Riacho da OB, Rambo da Mundo Novo, Zefec Abdala, Rapilho da SI, Fajardo da GB, Nurmahal Col, Paysandu de Nav and Voltaire TE J

Abbreviation: G0, model without UPG and MF.

When **G** accounted for patriarch‐specific allelic frequencies (G3), it was constructed according to Lourenco et al. (2016) as follows:

(7) $$G=ZZ^{\prime },\text{with}\;Z=\left(M-P_{k}\right)/\left[2\sum_{j=1}^{m}p_{\mathrm{jk}}\left(1-p_{\mathrm{jk}}\right)\right]$$

where M is the gene content matrix; $P_{k}$ contains twice the allele frequency of the *k*th MF; and $p_{\mathrm{jk}}$ is the allele frequency at the *j*th locus for the *k*th MF. It is worth noting that when allele frequencies are considered homogeneous across MF, this results in the **G** as in Vanraden (2008).

### 2.4 Evaluation of Model Performance

Scenarios were compared using linear regression (Legarra and Reverter 2018) method. The whole dataset (represented by subscript w) included all sources of information for all animals (records from 1970 to 2021). Two partial datasets were created, removing either the last 2 years of data (only for SC365 and AFC) or the last 3 years of data (only for ACP). This was done to ensure enough number of focal animals for the validation. Focal animals were young, genotyped individuals without their own (or progeny) records in the partial dataset (Table 3). The whole dataset can be interpreted as the posterior confirmation of the validity of the selection decisions. In contrast, the partial dataset represents the evaluation conducted during selection decisions (Macedo et al. 2020).

**TABLE 3:** Number of records in the whole and partial datasets and validation animals used in LR method for the different scenarios for longevity and reproductive traits in Nellore breed.

	SC365	AFC	ACP
Whole	239.806	560.785	269.330
Partial	222.318	546.595	251.266
Validation^a^	17.488	14.190	18.064
Years of validation	2020–2021	2019–2020	2017–2019

^a^ Young animals with genotypes and phenotypes.

The validation statistics were accuracy, bias, dispersion and correlation, computed as follows:

(8) $$\text{accuracy}=\hat{\mathrm{acc}}=\sqrt{\frac{\mathrm{cov}\left({\hat{u}}_{w},{\hat{u}}_{p}\right)}{\left(1-\bar{F}\right){\hat{\sigma }}_{u}^{2}}}$$

(9) $$\text{bias}={\hat{∆}}_{\mathrm{wp}}=\frac{{\bar{\hat{u}}}_{p}-{\bar{\hat{u}}}_{w}}{\sigma_{u}}$$

(10) $$\text{dispersion}={\hat{b}}_{w,p}=\frac{\mathrm{cov}\left({\hat{u}}_{w},{\hat{u}}_{p}\right)}{\mathrm{var}\left({\hat{u}}_{p}\right)}$$

(11) $$\text{correlation}=\text{corr}=\frac{\mathrm{cov}\left({\hat{u}}_{w},{\hat{u}}_{p}\right)}{\sqrt{\mathrm{var}\left({\hat{u}}_{w}\right)\mathrm{var}\left({\hat{u}}_{p}\right)}}$$

where $\mathrm{cov}\left(∙\right)$ is the sample covariance; ${\hat{u}}_{w}$ is the vector of GEBV from the whole dataset; ${\hat{u}}_{p}$ is the vector of GEBV from the partial dataset; $\bar{F}$ is the average pedigree‐inbreeding coefficient for focal animals; ${\hat{\sigma }}_{u}^{2}$ is the estimated additive genetic variance; ${\bar{\hat{u}}}_{p}$ is the average of ${\hat{u}}_{p}$ (likewise for ${\bar{\hat{u}}}_{w}$) and $\mathrm{var}\left(∙\right)$ is the sample variance.

## 3 Results and Discussion

### 3.1 Variance Components and Heritability

Variance components and genetic parameters are presented in Table 4. Direct heritability for SC365 (0.40) was similar to those reported by Kluska et al. (2018) (0.48), Silva Neto et al. (2020) (0.33), Carvalho Filho et al. (2020) (0.47) and Negreiros et al. (2022) (0.36), suggesting that it is possible to achieve genetic progress when selecting for SC365. Additionally, the heritability estimates for AFC (0.07) and ACP (0.12) indicate a larger environmental effect on these traits, supporting prior research on Nellore cattle (Kluska et al. 2018; Silva Neto et al. 2020; Negreiros et al. 2024).

**TABLE 4:** Variance components estimates and heritabilities for longevity and reproductive traits in Nellore breed.

Traits	$\sigma_{d}^{2}$	$\sigma_{e}^{2}$	$h_{d}^{2}$ ± SD
SC365	1.24	1.87	0.40 ± 0.08
AFC	1.41	17.81	0.07 ± 0.03
ACP	72.68	551.74	0.12 ± 0.05

Abbreviations: $\sigma_{d}^{2}$, additive variance in liability scale; $\sigma_{e}^{2}$, residual variance in liability scale.

Moreover, our heritability estimates were consistent with those found in other studies. The relatively low heritability observed, particularly in reproductive traits, may be attributed to factors such as population structure, the statistical model employed for variance component estimation and the gradual reduction of genetic variance in populations subjected to selection (Hidalgo et al. 2020).

### 3.2 Relationship Within and Across MF (Γ)

Within‐group relationships (diagonal of Γ) were smaller than one, while between‐group relationships (off‐diagonal of Γ) were different from zero across all three scenarios (S1, S2 and S3). In Γ_1_, relationships within the group ranged from 0.71 to 0.72, whereas between the two groups, it was 0.70. Γ_2_ represents the relationships for S2, with within‐group values ranging from 0.70 to 0.73 and a between‐group value of 0.70. Furthermore, Γ_3_ depicts the relationships for S3, with within‐group values ranging from 0.71 to 0.77 and between‐group values ranging from 0.68 to 0.72:

(12) $$\Gamma_{1}=\left[\begin{array}{cc} 0.71 & 0.70\\ \text{symm}. & 0.72 \end{array}\right]$$

(13) $$\Gamma_{2}=\left[\begin{array}{cc} 0.73 & 0.70\\ \text{symm}. & 0.70 \end{array}\right]$$

(14) $$\Gamma_{3}=\left[\begin{array}{cccccccccc} 0.77 & 0.69 & 0.71 & 0.70 & 0.72 & 0.72 & 0.71 & 0.72 & 0.71 & 0.70\\ & 0.71 & 0.69 & 0.69 & 0.70 & 0.69 & 0.69 & 0.69 & 0.69 & 0.68\\ & & 0.72 & 0.70 & 0.71 & 0.71 & 0.71 & 0.71 & 0.70 & 0.69\\ & & & 0.71 & 0.70 & 0.70 & 0.69 & 0.70 & 0.70 & 0.69\\ & & & & 0.73 & 0.71 & 0.71 & 0.71 & 0.71 & 0.69\\ & & & & & 0.74 & 0.71 & 0.71 & 0.70 & 0.70\\ & & & & & & 0.75 & 0.71 & 0.70 & 0.70\\ & & & & & & & 0.74 & 0.71 & 0.69\\ & & & & & & & & 0.72 & 0.69\\ \text{symm}. & & & & & & & & & 0.72 \end{array}\right]$$

The values observed on the diagonal are considered high because the individuals within MF share many alleles identical by descent (IBD). Additionally, the off‐diagonal values indicate that the MF also share a significant proportion of their genetic base, as evidenced by their highly similar allele frequencies. This pattern can be attributed to the origin of the first animals imported from India, which gave rise to the patriarchs used as MF. These animals likely descended from a limited number of founders, resulting in a narrow genetic base.

In commercial and registered herds, there is commonly an intense genetic flow between these groups due to the shared use of sires and reference genetics. This interconnection reduces differences in allele frequencies among MF, thereby increasing their genetic correlation, as indicated by the off‐diagonal values. Finally, regarding individuals with unknown paternity, even though they lack parental information in the pedigree, they belong to the same general population. As a result, they share a significant proportion of allele frequencies with other MF, which explains the high correlation values observed off the diagonal.

Legarra et al. (2015) discussed the significance of relationships within MF, where a value less than one suggests negative inbreeding, indicating divergence from the base population. Conversely, values exceeding one denote inbreeding within the base population, reflecting a higher degree of relatedness. Positive relationships observed across MF pairs imply overlapping ancestor populations. On the other hand, negative values signify population divergence, highlighting distinct genetic lines. Moreover, the base populations are unrelated when the relationship between MF pairs equals zero. In genetic evaluations, a gamma value different from zero between MF is crucial for incorporating MF into the system. This is because a gamma equal to zero implies that MF is equal to UPG, as highlighted by Bradford et al. (2019) and Kluska et al. (2021).

We identified substantial relationships within and across MF, indicating a robust association significantly different from zero. Bradford et al. (2019) illustrated relationships within MF ranging from 0.54 to 0.71 in a simulated dairy cattle population. Similarly, Legarra et al. (2015) observed relationships of 0.55 and 0.77 in Holstein and Jersey breeds, respectively. Exploring the relatedness of parental lines, van Grevenhof et al. (2018) uncovered correlations between MF of 0.17 and 0.74. Kluska et al. (2021) conducted a study with the Montana composite cattle, revealing relationships within four and ten MF, ranging from 0.15 to 0.38 and 0.15 to 0.65, respectively. Furthermore, relationships across MF ranged from 0.09 to 0.18 and − 0.11 to 0.23. Negative values in a composite population are expected once they indicate genetic divergence from the base. In our study, regardless of the MF definition, a strong association between the different populations was found. This could be interpreted as commercial and registered populations being genetically one single population or that regarding the use of different genetic lines (i.e., patriarchs) there is no different base in the population. Under this reasoning, little to no effect of MF in the validation statistics could be expected since treating the entire population as having a single base seems to be adequate.

### 3.3 Validation

**FIGURE 1:**
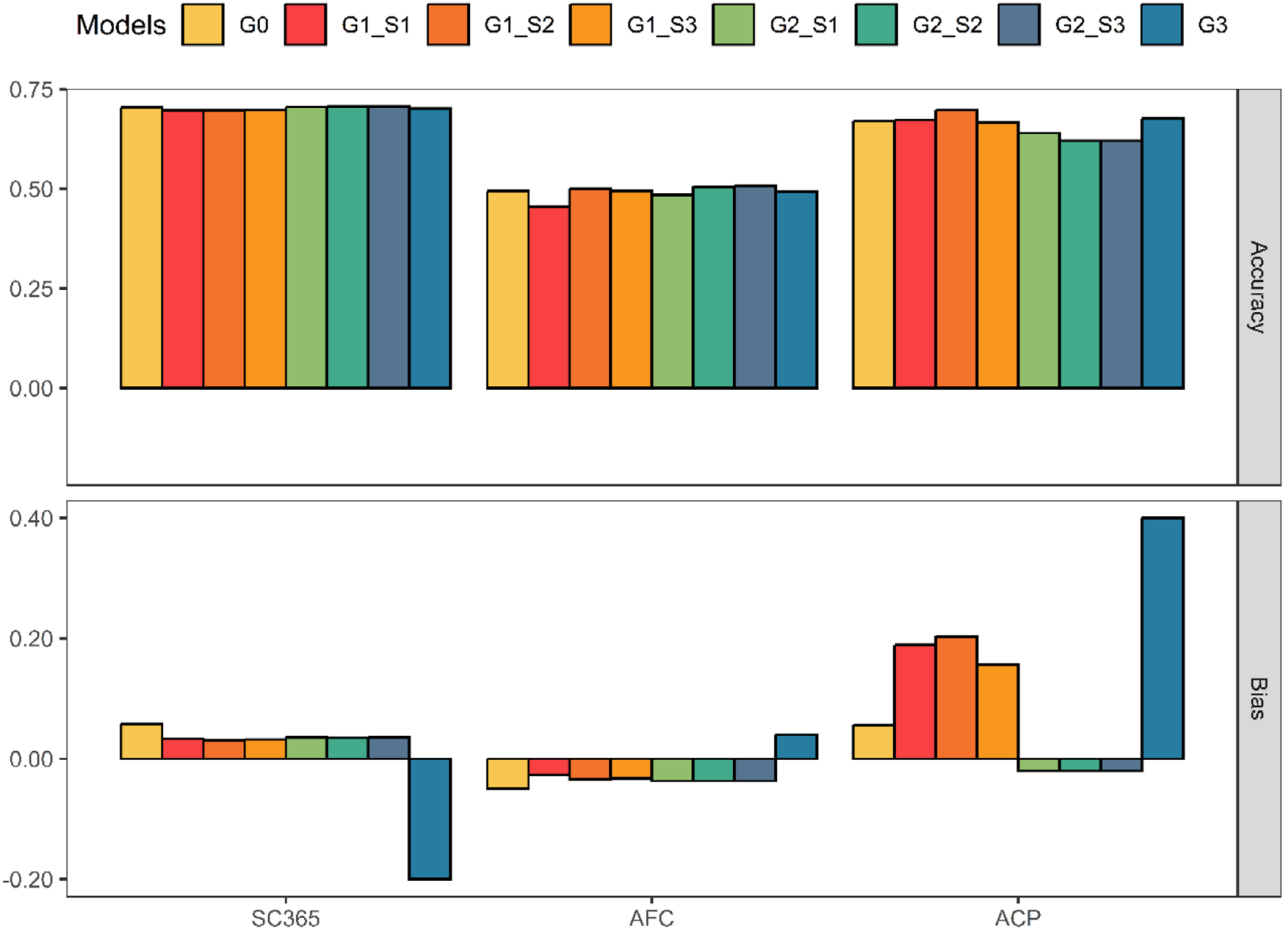
Accuracy and bias for genomic predictions of the validation animals according to the studied trait. Abbreviations: ACP, accumulated cow productivity; AFC, age at first calving; G0, without MF and UPG; G1, MF; G2, UPG; G3, G accounted for patriarch‐specific allele frequencies; S0, without MF and UPG; S1, commercial and registered herd; S2, paternity; S3, patriarchs; SC365, scrotal circumference. [Colour figure can be viewed at wileyonlinelibrary.com]

Accuracy and bias of genomic predictions for the validation animals in each scenario and trait are shown in Figure 1 and Table 5. An effective model must be capable of accurately predicting breeding values or future phenotypes, as this accuracy is essential for achieving genetic gain. The G0 model resulted in an accuracy of 0.70 (SC365), 0.49 (AFC) and 0.67 (ACP). Among these, AFC showed a decrease in the model G1_S1_ (0.45) incorporating MF compared to G0. The accuracy for SC365 remained consistent at 0.70 across all three MF models.

**TABLE 5:** Accuracy and bias of non‐genotyped and genotyped animals comparing the ssGBLUP predictions between models and traits.

Models	SC365		AFC		ACP	
	Acc	*δ*	Acc	*δ*	Acc	*δ*
G0	0.70	0.06	0.49	−0.05	0.67	0.06
G1S1	0.69	0.03	0.45	−0.03	0.67	0.19
G1S2	0.69	0.03	0.50	−0.03	0.70	0.20
G1S3	0.69	0.03	0.49	−0.03	0.67	0.16
G2S1	0.70	0.04	0.48	−0.04	0.64	−0.02
G2S2	0.70	0.04	0.50	−0.04	0.62	−0.02
G2S3	0.70	0.04	0.51	−0.04	0.62	−0.02
G3	0.70	−0.20	0.49	0.04	0.68	0.40

Abbreviations: δ, bias; acc, accuracy; ACP, accumulated cow productivity; AFC, age at first calving; G0, without MF and UPG; G1, MF; G2, UPG; G3, G accounted for patriarchs MF specific allele frequency; S1, commercial and registered herd; S2, paternity; S3, patriarchs; SC365, scrotal circumference at 305 days.

The model G1_S2_ led to improved accuracy for both AFC (0.50) and ACP (0.70). The accuracy for AFC and ACP in the model G1_S3_, as well as ACP in the model G1_S1_, remained unchanged with the inclusion of MF. When considering the use of UPG relative to the model G0, SC365 remained stable and AFC decreased to 0.48 in the model G2_S1_, and ACP decreased in all models to 0.62. In the G3 model, where patriarch‐specific allelic frequencies were accounted for **G**, there was an increase in accuracy for ACP (0.68), while the accuracy for SC365 and AFC remained unchanged.

Several studies have highlighted the advantages of using MF in genomic evaluations. Bradford et al. (2019) found a significant increase in accuracy when using MF compared to the UPG in a dairy cattle population. Similar results were observed by Kudinov et al. (2022) in red dairy cattle and by Macedo et al. (2020) in sheep. Londoño‐Gil et al. (2024), employing a multi‐racial approach (Nellore, Brahman, Guzerat and Tabapua) and Kluska et al. (2021) working with Montana composite cattle found considerable increases in accuracy when using MF for weight and SC365. Garcia‐Baccino et al. (2017) and van Grevenhof et al. (2018) tested models with and without MF and found a small or no difference in accuracy.

The contribution of **G** to the breeding value estimates is reduced when the number of genotyped animals is limited, and the increase in accuracy with genomic information tends to be modest (Lourenco et al. 2015). The accuracy of genomic predictions is influenced by the number of genotyped animals connected with MF, the quantity of markers, the size of training and validation, and heritability (Gondro et al. 2013; Cesarani et al. 2021; Melo et al. 2024).

Even though the models are still accurate, our results suggest that using MF may not increase the accuracy of GEBV. According to Bradford et al. (2019), the accuracy of GEBV is more related to the trait's heritability than to the inclusion of MF or UPG in ssGBLUP. It was anticipated that traits with high heritability would exhibit an improvement in prediction accuracy, as observed by Bradford et al. (2019). Using simulated data for a trait with a heritability of 0.30, they reported an improvement in accuracy from 0.36 to 0.77 when applying ssGBLUP with MF, when compared to models without correction for missing pedigree. However, our results did not confirm this trend. Interestingly, two traits with low heritability, AFC and ACP, showed significant benefits. Bradford et al. (2019) also demonstrated, through simulated data for a trait with a heritability of 0.10 (considered low), that the MF model using ssGBLUP was among the best performers, yielding an accuracy of 0.64 and a bias of −0.01.

Low‐heritability traits generally respond less effectively to direct selection due to a lower proportion of additive genetic variance. However, the use of methodologies such as MF and UPG enhances the capture of information on the animals' genetic origins, enabling bias correction in GEBV. Additionally, by introducing MF, a relationship is assumed among founders that share similar genes, which facilitates the capture of additional variability and provides a more robust foundation for traits with challenging genetic prediction, such as AFC and ACP. This network of related founders contributes to a more consistent and robust genetic evaluation across generations.

Model G0 resulted in no bias for all studied traits (0.06 for SC365, −0.05 for AFC and 0.06 for ACP). Models using MF for SC365 and AFC or UPG for SC365, AFC and ACP presented bias close to zero, ranging from −0.02 to 0.04. Negative bias indicates an underestimation of GEBV (Legarra and Reverter 2018; Kluska et al. 2021) and were found for AFC using models with MF and UPG. According to Bradford et al. (2019), animals are more impacted by UPG when recent pedigree information is lacking, as $A_{22}^{-1}$ reflects only known pedigree relationships, whereas the inverse of **G** accounts for all genetic relationships, even in the absence of complete pedigree data. Therefore, it was expected that models incorporating MF would exhibit less bias compared to those using UPG. The highest values of bias were found for ACP, showing an increase from −0.02 to 0.20 between models with UPG and MF. The highest bias for ACP was observed in model G3 (0.40), which used the G matrix centred on the allele frequencies of the MF. Similarly, model C3 resulted in greater bias for SC365 (−0.20). In the context of genomic prediction, the ideal scenario is to obtain bias values close to zero, indicating that predicted values are neither systematically overestimated nor underestimated. The increase observed with the use of MF may be attributed to an insufficient number of genotyped animals to accurately estimate MF effects, particularly since the number of genotypes and phenotypes varied across traits and with the dataset structure.

Another possible explanation for the increased bias in ACP could be selective phenotyping, considering the trait's low heritability and the fact that not all animals have available data, as females must complete the production cycle to be phenotype. This trait is not directly measured but is based on a calculation that includes the calving index, number of calves and weaning weight during the female's time in the herd. Additionally, it depends on the AFC, the interval between calvings and how long the cows stay in the herd (Lôbo et al. 2000).

Overall, compared to the G0 model, MF helped reduce bias for SC365 and AFC, which are less influenced by selective phenotyping, whereas UPG benefited all traits. However, using MF in the multi‐trait model increased the bias for ACP. In the G3 model (highest bias for SC365 and ACP), which incorporates patriarch‐specific allele frequencies into the matrix **G**, an increase in bias was observed for both SC365 (−0.20) and ACP (0.40). This increase in bias could be attributed to the effects of allele frequency weighting, which may fluctuate due to genetic drift and selection. When allele frequencies in the base population are accounted for, particularly under the assumption that founders in this population are non‐genotyped but connected through pedigree information, these effects can become more pronounced (Neshat et al. 2023). The same authors also noted that inaccuracies in pedigree information can make it challenging to accurately trace founders and establish the true base allele frequencies. However, genotypic data can still capture substantial information about these frequencies, providing a more reliable estimate than pedigree data alone, though the absence of precise pedigree records may still introduce some uncertainty. Based on the results obtained and in comparison with findings from the literature, defining MF based on patriarchs may not improve accuracy and could increase the bias, especially for ACP, as observed in the G3 model where the patriarch‐specific allele frequencies were used.

Londoño‐Gil et al. (2024) found an increase in bias for weight at 450 days for Brahman and Guzerat, as well as SC365 for all breeds when using MF. The authors also mentioned that if the animals are related solely through **A**_**22**_ rather than **G**, the mismatch between these matrices can introduce bias and result in a loss of accuracy. In contrast to our findings, Bradford et al. (2019), in a simulation study, found an increase in bias in pedigree‐based models when UPG were used to fill in pedigree gaps. Kluska et al. (2021), working with the Montana composite beef cattle, found that including UPG in pedigree‐based models resulted in higher bias. The authors explained that the complex population structure is the main factor causing bias. In general, lower bias estimates indicate that MF and UPG effectively capture the population's genetic structure. This robustness suggests the model can accurately manage incomplete pedigree information, leading to more accurate genetic evaluations. It is important to highlight that the positive or negative effects observed from the different methods are highly influenced by the data structure, which should be considered when interpreting the results (Himmelbauer et al. 2024). This explains why different studies reached different conclusions about the same method (Bradford et al. 2019).

**FIGURE 2:**
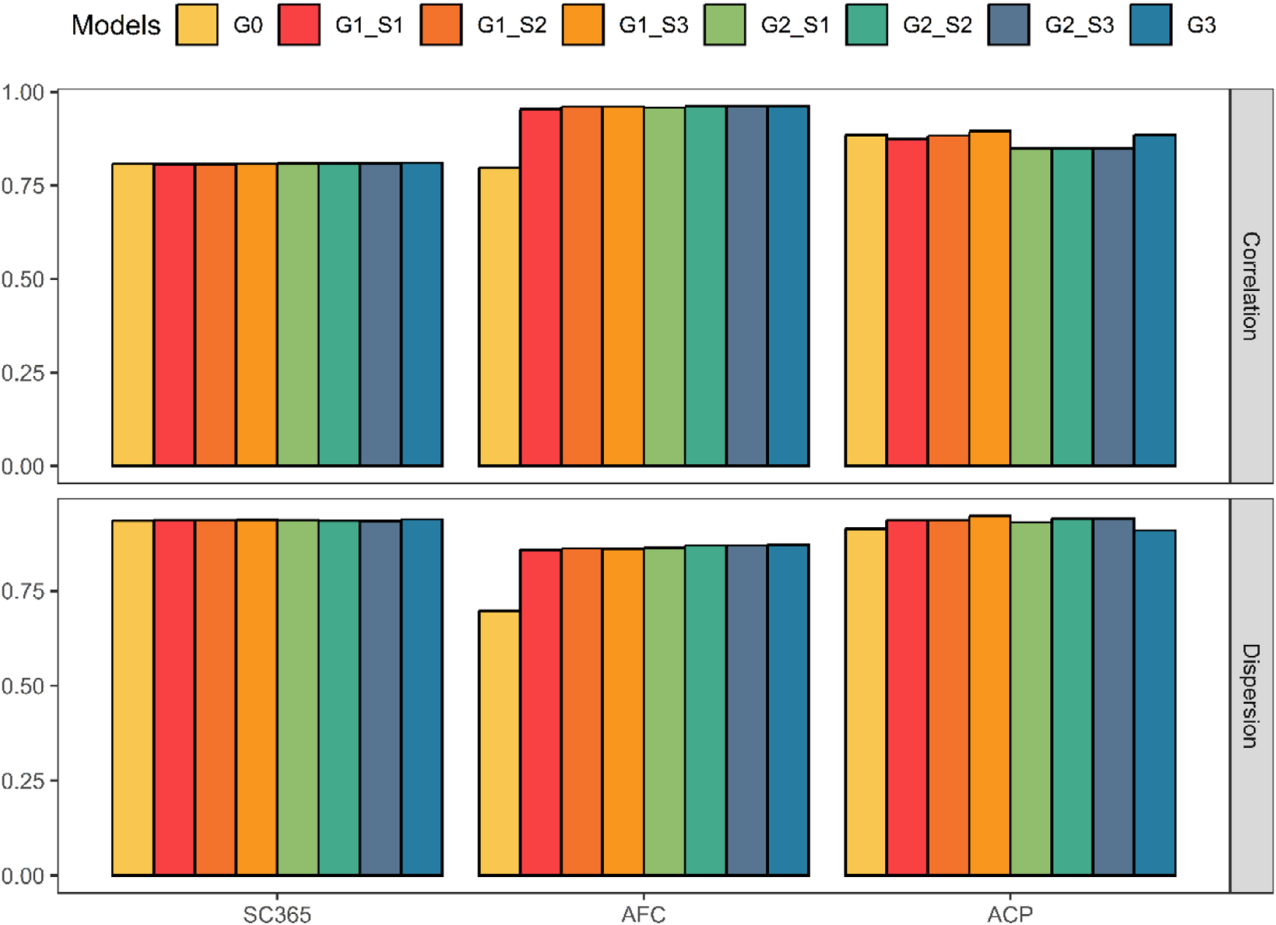
Correlation and dispersion for genomic predictions of the validation animals according to the studied trait. Abbreviations: ACP, accumulated cow productivity; AFC, age at first calving; G0, without MF and UPG; G1, MF; G2, UPG; G3, G accounted for patriarchs MF specific allele frequency; S0, without MF and UPG; S1, commercial and registered herd; S2, paternity; S3, patriarchs; SC365, scrotal circumference. [Colour figure can be viewed at wileyonlinelibrary.com]

Correlation between GEBV from different models, as well as dispersion, is shown in Figure 2 and Table 6. The dispersion (i.e., slope), which can be observed, particularly when selection candidates span across various generations or possess varying degrees of information (Piccoli et al. 2018; Kluska et al. 2021). Correlations ranged from 0.81 to 0.96, whereas dispersion had a smaller amplitude, between 0.86 and 0.95.

**TABLE 6:** Correlation and dispersion of non‐genotyped and genotyped animals comparing the ssGBLUP predictions between models and traits.

Models	SC365		AFC		ACP	
	Corr	b1	Corr	b1	Corr	b1
G0	0.81	0.93	0.80	0.70	0.88	0.91
G1S1	0.80	0.94	0.95	0.86	0.87	0.94
G1S2	0.80	0.94	0.96	0.86	0.88	0.94
G1S3	0.81	0.94	0.96	0.86	0.89	0.95
G2S1	0.81	0.94	0.96	0.86	0.85	0.93
G2S2	0.81	0.93	0.96	0.87	0.85	0.94
G2S3	0.81	0.93	0.96	0.87	0.85	0.94
G3	0.81	0.94	0.96	0.87	0.89	0.91

Abbreviations: ACP, accumulated cow productivity; AFC, age at first calving; b1, dispersion; corr, correlation; G0, without MF and UPG; G1, MF; G2, UPG; G3, G accounted for patriarchs MF specific allele frequency; S1, commercial and registered herd; S2, paternity; S3, patriarchs; SC365, scrotal circumference at 305 days.

For the SC365 trait, the models show similar correlations, ranging from 0.80 to 0.81, and dispersion between 0.93 and 0.94. This pattern suggests that the inclusion of MF or UPG does not bring significant changes in accuracy for this trait. This stability may be attributed to the nature of this trait, which appears less dependent on different relationship parameterisations. For AFC, a more significant impact of modelling MF and UPG effects is observed, with correlations ranging from 0.80 (G0) to 0.96 (G1_S2_, G1_S3_, G2_S1_, G2_S2_, G2_S3_ and G3). These results indicate that the inclusion of MF or UPG substantially improves predictive accuracy for AFC, possibly due to the higher genetic and environmental complexity associated with this reproductive trait. The dispersion for AFC also increases in models with MF or UPG, rising from 0.70 in G0 to up to 0.87 in G3, suggesting an improvement in the match between predictions and observed values. For ACP, the correlation is high across all models, with slight variations between 0.85 (G2_S1_, G2_S2_, G2_S3_) and 0.89 (G1_S3_ and G3). However, models with lineage‐specific MF (G1_S3_ and G3) show the highest correlations, suggesting that including information on lineage‐specific alleles can capture important genetic nuances for accumulated productivity. The correlations obtained between GEBV from the whole dataset and GEBV from the partial dataset are essential to understanding the efficiency and robustness of genomic prediction models. High correlations suggest that the prediction model captures genetic variability well and may be effective in predicting traits in the validation population. High correlations indicate that the model is transferable between populations and that the training captured relevant genetic information. Low correlations may suggest that the model is overfitting to the training data and does not generalise well to the validation population. Thus, this parameter is important to indicate whether genomic prediction can effectively select superior and inferior individuals, demonstrating the model's usefulness. Regardless of the approach used, the estimates of correlations between the GEBVs of the complete and partial populations obtained demonstrate that the models can be used for genomic prediction of SC365 and AFC.

Kluska et al. (2021) showed that including genomic information helped reduce inflation for the weaning weight trait in the Montana composite cattle. However, in this study, the overall impact using UPG or MF for dispersion was low. Bradford et al. (2019) noted that dispersion for BLUP models without considering the absence of pedigrees may be greater than in models with genomics. Kluska et al. (2021) found a lower correlation between genomic models than between BLUP models, and the correlation was higher between BLUP models and ssGBLUP_MF_. The authors also commented that if animals with and without phenotypes in the same group are unrelated, group effects are not estimable and the model is similar to ignoring UPG. Additionally, if genotyped animals are unrelated to non‐genotyped animals, **H** ^−1^ will not contribute to estimating the group solution, and it will be similar to ignoring UPG (Tsuruta et al. 2019). In single‐breed evaluations, Londoño‐Gil et al. (2024) observed minor changes in Pearson correlations between non‐genotyped and genotyped animals. They explained that this occurs because alterations in **G** and **A**_22_ directly affect these individuals based on the propagation of genomic information to non‐genotyped animals in the **H** matrix. The dispersion is relatively stable across models, ranging from 0.91 to 0.95, indicating good consistency in prediction for this trait. Regarding correlations, higher values are associated with greater precision in the model when identifying genetically superior animals. This precision facilitates selecting individuals with superior reproductive performance, promoting accelerated genetic progress, which is crucial for traits of high economic relevance, such as those related to reproduction and longevity.

Improvement in EBVs can be observed through increased prediction accuracy by including additional phenotypic information and molecular marker data. In the present study, the criteria for defining MF and UPG did not significantly improve over the default model, particularly for patriarchs. This aspect interested producers, who wanted to assess whether including patriarchs classification would benefit genetic evaluations. These results may be attributed to the limited number of genotyped and phenotyped animals associated with these patriarchs. With increased phenotypes, genotypes and pedigree information related to one another, greater accuracy and reduced bias would likely be evident. Additionally, the criteria chosen for defining MF and UPG may not have been the most suitable for the studied traits, as the literature generally uses the year of birth, animal sex, country of origin and breed as the basis for establishing MF and UPG.

## 4 Conclusion

MF and UPG provided similar GEBV for all traits, with no significant increase in prediction accuracy. Dispersion and correlation remained close to one, indicating no inflation or deflation in the GEBV for younger animals, regardless of the methodology used. Centring and scaling **G** by the allelic frequency of the patriarchs yielded similar accuracy and bias compared to MF, except for APC, which showed higher bias, possibly due to the selective phenotyping. Over time, adding more genotypes and phenotypes to the database has the potential to improve estimates using MF, particularly for reproductive, longevity and productivity traits in Nellore cattle.

## Ethics Statement

The authors have nothing to report.

## Conflicts of Interest

The authors declare no conflicts of interest.

## Data Availability

The data that support the findings of this study are available from the National Association of Breeders and Researchers (ANCP). The data sets generated and/or analysed during the current study are available through the corresponding author upon reasonable request with the permission of the National Association of Breeders and Researchers (ANCP).
